# High density of tryptase-positive mast cells in human colorectal cancer: a poor prognostic factor related to protease-activated receptor 2 expression

**DOI:** 10.1111/jcmm.12073

**Published:** 2013-08-29

**Authors:** Andrea Malfettone, Nicola Silvestris, Concetta Saponaro, Girolamo Ranieri, Antonio Russo, Stefano Caruso, Ondina Popescu, Giovanni Simone, Angelo Paradiso, Anita Mangia

**Affiliations:** a Functional Biomorphology Laboratory, National Cancer Research Centre “Giovanni Paolo II”Bari, Italy; b Medical Oncology Unit, National Cancer Research Centre “Giovanni Paolo II”Bari, Italy; c Interventional Radiology Unit, National Cancer Research Centre “Giovanni Paolo II”Bari, Italy; d Section of Medical Oncology Department of Surgical, Oncological and Stomatological Sciences University of PalermoPalermo, Italy; e Pathology Department, National Cancer Research Centre “Giovanni Paolo II”Bari, Italy; f Scientific Direction, National Cancer Research Centre “Giovanni Paolo II”Bari, Italy

**Keywords:** Colorectal cancer, PAR-2, mast cell, tryptase, NHERF1, prognostic factor, invasiveness, aggressiveness

## Abstract

Tryptase(+) mast cells (MCs), abundant in the invasive front of tumours, contribute to tissue remodelling. Indeed, protease-activated receptor-2 (PAR-2) activation by MC-tryptase is considered an oncogenic event in colorectal cancer (CRC). Recently, we have suggested NHERF1 as a potential new marker in CRC. In this study, we aimed to determine the distribution of tryptase(+) MCs and PAR-2 and to examine the relationship between PAR-2 and NHERF1, investigating their reputed usefulness as tumour markers. We studied a cohort of 115 CRC specimens including primary cancer (C) and adjacent normal mucosa (NM) by immunohistochemical double staining, analyzing the protein expression of MC-tryptase, PAR-2 and cytoplasmic NHERF1. MC density was higher in NM than in C. Tumours with high TNM stage and poor grade showed the highest MC density. A higher PAR-2 immunoreactivity characterized tumours most infiltrated by MCs compared with samples with low MC density. Furthermore, PAR-2 overexpression was associated with advanced TNM stage, poor grade and lymphovascular invasion (LVI). A positive correlation existed between tryptase(+) MC density and PAR-2 expression. Cytoplasmic NHERF1 was higher in C than in NM and overexpressing tumours resulted associated with nodal and distant metastases, poor grade and LVI. PAR-2 correlated with cytoplasmic NHERF1 and the PAR-2(+)/cytoplasmic NHERF1(+) expression immunophenotype identified tumours associated with unfavourable prognosis and aggressive clinical parameters. Our data indicate that the high density of tryptase(+) MCs at invasive margins of tumours was associated with advanced stages of CRC and was strongly correlated with PAR-2 expression.

## Introduction

Cancer-related inflammation is a key component of tumours and has been suggested as the seventh hallmark of cancer progression [1]. In fact, patients with inflammatory bowel disease, Crohn's disease and chronic ulcerative colitis have a much higher risk of CRC [2, 3]. MCs play a role as cancer promoters through various molecular and cellular mechanisms, including participation in immunosuppression, release of pro-angiogenic and mitogenic factors and remodelling of the tumour microenvironment [4–6]. MCs contribute to tissue remodelling through a rich number of proteases, including tryptase. Indeed, tryptase(+) MCs have been found to be abundant in the invasive front of tumours [7].

It is well-established that a subclass of proteases acts as signal molecules controlling cell functions through PARs, a subfamily of specific G protein–coupled receptors consisting of four members designated PAR1-4 [8]. In particular, PAR-2 is activated by multiple trypsin-like enzymes including trypsin and MC-tryptase, contributing to a range of normal and disease processes including cancer. Activation of PAR-2 is an oncogenic event in colon cancer [9] and it may facilitate the progression of CRC [10, 11]. MC-tryptase cleaves and activates PAR-2 on gut epithelial cells and myocytes [12, 13]. PAR-2 activity also stimulates proliferation of colon cancer cells and induces the expression of the prostaglandin-synthesizing enzyme COX2 and its metabolite prostaglandin-E2 [14]. Moreover, recent studies have shown that the PAR-2/trypsin system facilitates colorectal carcinogenesis by promoting cell proliferation, migration and invasion [15].

Signalling pathways are assembled into multi-protein signalling complexes by scaffolding proteins that permit the creation of new tumour-specific pathways to drive the subverted cellular functions exhibited by tumour cells [16]. The Na^+^/H^+^ exchanger regulating factor 1 (NHERF1, also named SLC9A3R1) is an adaptor protein that functions as a regulator of transmembrane receptors, transporters and other proteins localized at or near the plasma membrane [17] and is considered a new player in CRC progression [18]. Recently, we have suggested that nuclear NHERF1 could be a potential new marker, representing an early indicator of pre-morphological triggering of CRC carcinogenesis [19] and demonstrating an association with the hypoxic tumour microenvironment in nodal and liver metastases [20].

This study examined the distribution of tryptase(+) MCs and the expression of PAR-2 in primary tumours compared with adjacent uninvolved mucosa, in an attempt to understand their role in the progression of colonic cancer. Moreover, we examined protein expression of NHERF1 and its relationship with PAR-2, with the aim to investigate their supposed usefulness as tumour markers in CRC.

## Materials and methods

### Ethics statement

The study was approved by the Institutional Review Board of the National Cancer Research Centre ‘Giovanni Paolo II’, Bari, Italy. Before undergoing routine surgery, all patients were informed of the investigational nature of the study and provided their written informed consent, authorizing the Institute to utilize their removed biological tissue according to ethical standards.

### Tissue specimens

A series of 115 colonic cases of pathologically confirmed primary adenocarcinoma Not Otherwise Specified (NOS) matched with adjacent cancer-uninvolved colonic mucosa was identified by a retrospective search of the surgical pathology files of the Pathology Department of our Institute.

Sections of 4-μm thickness were cut from formalin-fixed and paraffin-embedded histological blocks and for each sample, the cancer (C) and the paired NM present on the same slide were used for comparison. Histopathologic features of the colon carcinoma tissue specimens were confirmed by blinded review of the original pathology slides, stained with routine haematoxylin and eosin.

Disease was staged according to the tumour-node-metastasis (TNM) classification system of the American Joint Committee on Cancer [21]. The World Health Organization classification was used for pathologic grading [22]. Assessment of LVI was based on examination of sections stained with haematoxylin and eosin and was considered evident if at least one cohesive clump of tumour cells was clearly visible within peritumoural endothelial-lined spaces, both lymphatic channels and small blood vessels—closely associated with primary invasive carcinoma [23].

Information regarding patient characteristics, including age, gender, histological type, tumour site, depth of invasion, nodal status, distant metastases, differentiation grade, LVI, TNM stage and EGFR status, was collected from the Pathology Department of our Institute. The EGFR status was realized in accordance with the EGFR pharmDx™ scoring guidelines. Results were reported as positive when a complete or incomplete circumferential membrane staining was observed in at least 1% of the tumour cells. Staining was defined as immunostaining of cell membranes above background level and scored as follows: 1+=weak, 2+=moderate and 3+=strong. The absence of membrane staining or cytoplasmic staining was reported as negative. The percentage of stained cells was assessed as follows: 1–10, 10–50 and >50%. Slides were scored in a blind manner by two independent observers, who were blinded to the patients' characteristics. In the case of disagreement, the EGFR status was determined by consensus after simultaneous dual re-examination.

### Immunohistochemistry and immunohistochemical double staining

Sections were immunohistochemically stained for NHERF1 using standard immunoperoxidase techniques, as previously described [24]. Briefly, sections were deparaffinized with xylene, rehydrated through a graded ethanol series and pre-treated with 0.01 M sodium citrate buffer at pH 6.0 in water bath. After endogenous peroxidase activity blocking with 0.3% H_2_O_2_ buffer solution, sections were incubated with a rabbit polyclonal EBP50 antibody for NHERF1 (clone PA1-090; Affinity Bioreagents, Golden, CO, USA; 1:150 dilution in PBS/BSA 1%) overnight at 4°C. Bound antibody was visualized with 3-amino-9-ethylcarbazole substrate-chromogen (DakoCytomation, Glostrup, Denmark) in the dark and counterstained with Mayer's haematoxylin. As a positive internal control, we used paraffin-embedded cells pellets from MCF-7 cell lines, expressing high levels of NHERF1. For negative controls, the primary antibody was omitted and replaced by PBS pH 7.6.

Every serial section adjacent to that stained for NHERF1 was submitted to immunohistochemical double staining, a procedure that combined the two markers with the aim to analyse both density of MCs and the expression of PAR-2. Following the epitope antigen retrieval with 0.01 M sodium citrate buffer at pH 6.0 in water bath, protein blocking was performed with Tris-buffered saline pH 7.6/Bovine Serum Albumin (BSA) 1% and then the slides were exposed overnight to a mixture of antibodies directed against human MC-tryptase (clone FL-275, rabbit polyclonal; Santa Cruz Biotechnology Inc., Santa Cruz, CA, USA) and PAR-2 (clone SAM11, mouse monoclonal; Santa Cruz Biotechnology Inc.; for both 1:150 dilution in Tris-buffered saline/BSA 1%). After tissue rinsing in Tris-buffered saline 1× wash buffer, sections were incubated with MACH 2 Double Stain (Polymer Detection Kit 2, mouse-Horseradish Peroxidase + rabbit-Alkaline Phosphatase; Biocare Medical LLC, Concord, CA, USA) and bound antibodies were visualized with 3,3′-diamino-benzidine (DAB Chromogen Kit; Biocare Medical LLC) and Vulcan Fast Red chromogens (Vulcan Fast Red Chromogen Kit 2; Biocare Medical LLC). Finally, the slides were counterstained with Mayer's haematoxylin and mounted with an aqueous mounting medium (DakoCytomation). As a result, the staining with human MC-tryptase was visualized as a bright fuchsin-red colour, whereas the immunostaining of PAR-2 appeared as brown. In each assay, a section of CRC noted on routine histological examination with extensive infiltration by MCs and with the presence of muscularis mucosae served as positive control for MC-tryptase and PAR-2 respectively. As a negative control, the primary antibody was replaced with Tris-buffered saline/BSA 1%.

Immunohistochemical analysis for MCs was based on the appearance of tryptase(+) MCs in the tissue stroma, as previously reported [25]. To evaluate MC density, the stained sections were screened under ×50 microscopic fields to identify representative fields. Then, MCs were counted in six to eight non-overlapping ×200 microscopic fields, covering both peripheral and central regions (core) of each section. Cells showing an equivocal staining or lacking a nucleus were not counted. MC density in every histological section was expressed as a median and interval of variation, which was used as cut-off to classify cases in low (≤median) and high (>median) MC density.

Immunohistochemical analysis for PAR-2 and NHERF1 was based on subcellular localization of analysed markers. For PAR-2 immunoreactivity, membranous/cytoplasmic localization was considered, whereas for NHERF1, the cytoplasmic localization was examined. As previously described [20], protein expression analysis was determined by multiplying the number of stained cells (0–100) by the intensity staining (0, no immunoreactivity; 1, weak; 2, moderate; 3, strong) in three to five representative areas for each section at ×200 magnification. The total score obtained (0–300) was therefore converted to a percentage of stained positive cells/section. Moreover, the median value of positive cells was used as a cut-off to group cases into two categories of negative (≤median) and positive (>median) expression. All stained samples were scored in a blind manner by two independent investigators who had no prior knowledge of the clinicopathological data. When a section was uninformative—either lost or contained no tumour tissue—a case was judged as ‘not evaluable’ in the statistical analysis. Photomicrographs were acquired under bright-field illumination with a Leica DMLB optical microscope (Leica, Cambridge, UK), coupled with a Leica DCC camera (Leica).

### Immunohistofluorescence

Immunofluorescent analysis was performed as previously described [20]. Briefly, after antigen retrieval, tissues were incubated overnight at 4°C in a humidified chamber with the rabbit policlonal antibody anti-NHERF1 (clone PA1-090, 1:150 dilution; Affinity Bioreagents) and with the mouse monoclonal anti-PAR-2 (clone SAM11, 1:150 dilution; Santa Cruz Biotechnology Inc.). Slides were then incubated at room temperature for 1 hr with the Alexa Fluor 488 and Alexa Fluor 568 immunoglobulin G secondary conjugated antibodies (1:2000 dilution; Molecular Probes Inc., Eugene, OR, USA).

Images were obtained on a BX40 microscope (Olympus, Tokyo, Japan) with a SenSys 1401E-Photometrics charge–coupled device camera. To verify protein co-localization, each acquired stack was merged by transforming the two channels corresponding to red (tetramethylrhodamine B isothiocyanate) and green (fluorescein isothiocyanate) into a single two-colour stack using the ‘RGB merge’ command of ImageJ software (National Institutes of Health Bethesda, MD, USA).

### Statistical analysis

The non-parametric Mann–Whitney U-test was used to compare expression levels of tumour markers considered within C and NM compartments. Association analysis between tumour markers and clinicopathological data was analysed using a χ^2^ or Fisher's exact tests. Correlation between two continuous variables was assessed by the non-parametric Spearman's rank test. All tests were two sided with a 95% CI and a *P* < 0.05 was considered statistically significant. Data analysis was carried out using the statistical package SPSS 17.0 (SPSS Inc., Chicago, IL, USA).

## Results

### Patients

This study considered 115 patients, consisting of 66 men (57.4%) and 49 women (42.6%), with a median age of 66 years (range 40–89 years). Location of cancer was colon and rectum in 73 (64%) and 41 (36%) patients respectively. Sixty-two (62.6%) patients presented lymph node metastases at the time of diagnosis and 47 (45.6%) had synchronous distant metastases. Seven tumours (6%) were classified as well-differentiated, 57 (50%) as moderate and 50 (43.9%) as poorly differentiated. Based on the TNM stage classification, there were 18 (15.7%) stage I patients, 19 (16.5%) stage II patients, 27 (23.5%) stage III patients and 51 (44.3%) stage IV patients.

The clinicopathological characteristics of the 115 tumours analysed are summarized in Table 1.

**Table 1:** Clinicopathological data and tumour marker expressions in 115 colorectal cancers

Characteristics, *n* (%)	Total (*n* = 115)
Age at diagnosis, median (range)	66 (40–89)
Gender	
Male	66 (57.4)
Female	49 (42.6)
Histological type	
Adenocarcinoma (NOS)	115 (100.0)
Tumour site	
Colon	73 (63.5)
Rectum	42 (36.5)
Depth of invasion	
pT1	4 (3.5)
pT2	18 (15.7)
pT3	69 (60.0)
pT4	24 (20.9)
Nodal status	
pN0	43 (37.4)
pN1	32 (27.8)
pN2	37 (32.2)
pN3	3 (2.6)
Distant metastases	
M0	68 (59.1)
M1	47 (40.9)
Differentiation grade	
Well	7 (6.1)
Moderate	57 (50.0)
Poor	50 (43.9)
LVI	
Absent	60 (68.2)
Present	28 (31.8)
TNM stage	
I	18 (15.7)
II	19 (16.5)
III	27 (23.5)
IV	51 (44.3)
EGFR	
Negative	49 (73.1)
Positive	18 (26.9)
MC density	
Low (≤56)	59 (51.3)
High (>56)	56 (48.7)
PAR-2 expression	
Negative (≤26)	61 (53.0)
Positive (>26)	54 (47.0)
Cytoplasmic NHERF1 expression	
Negative (≤44%)	58 (50.4)
Positive (>44%)	57 (49.6)

Adenocarcinoma (NOS): adenocarcinoma Not Otherwise Specified; LVI: lymphovascular invasion; EGFR: epidermal growth factor receptor; MC density: density of tryptase(+) Mast Cell; PAR-2: Proteinase-Activated Receptor 2; NHERF1: Na^+^/H^+^ exchanger regulating factor 1.

### Protein expression analysis of tryptase(+) MCs and PAR-2

**Fig. 1:**
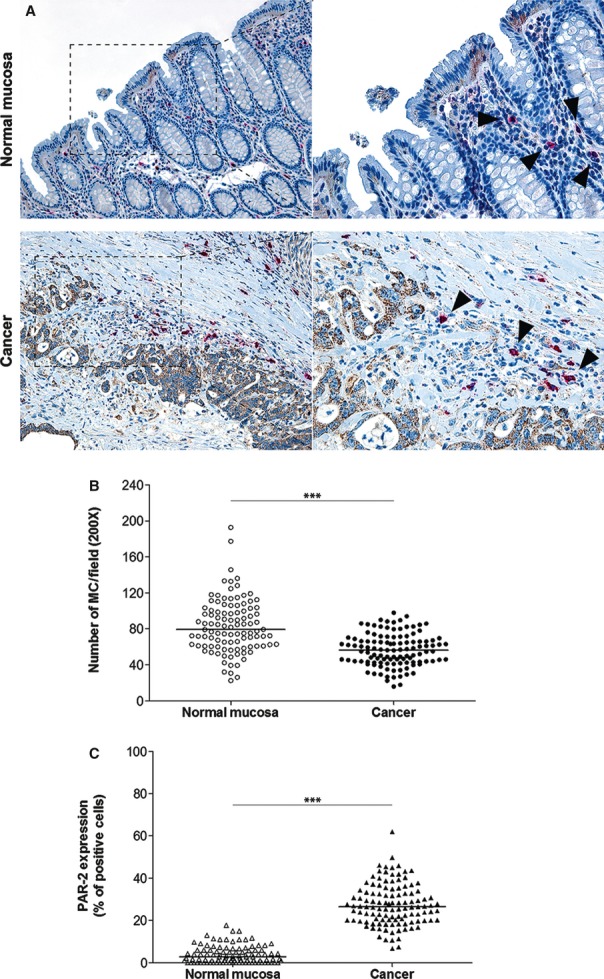
Expression analysis of tryptase-positive mast cells and PAR-2 in human colorectal cancer. (**A**) Representative images of tryptase(+) MCs (arrowheads) and PAR-2 immunoreactivity in primary tumours matched with adjacent cancer-uninvolved colonic mucosa by immunohistochemical double staining (original magnification on the left ×100, enlargement on the right ×200). (**B** and **C**) The MC density and the distribution of PAR-2(+) cells, respectively, on normal mucosa and the tumour compartment of the same colonic lesion. (Horizontal bold line in each box = median value; ***P < 0.0001).

We carried out an immunohistochemical double-staining assay to examine the distribution of mucosal MCs and to determine PAR-2 levels, obtaining bright fuchsine-red and brown-coloured precipitates at the antigen sites of cancer and corresponding normal mucosa compartments of the same colonic tumour respectively (Fig. 1A). Infiltration of MCs was already present in the uninvolved compartment of each sample, distributed in the lamina propria and submucosa (Fig. 1A, upper panel). Conversely, PAR-2 was lowly expressed or not detectable in epithelial cells of normal mucosa (Fig. 1A, upper panel).

In the tumour compartment, we observed MCs located mainly in the connective stroma, in the interface between growing cancer and healthy tissue and, frequently, in close association with small blood vessels within the tumour microenvironment. Interestingly, MCs were also present within the core of the invasive tumours and foci of microinvasion of the tumour mass (Fig. 1A, lower panel). In contrast, immunoreactivity for PAR-2 was detected both at the tumour centre and at the invasive front of the lesion. However, a higher intensity of staining could be detected along the invasion front and in most cases, along tumour borders adjacent to vascular structures (Fig. 1A, lower panel). In particular, PAR-2 was strongly expressed on cytoplasm or focally localized on membranous surface of epithelial tumour cells.

The distribution of MCs and PAR-2 immunoreactive epithelial cells in colonic tumours is summarized in Figure 1B and C respectively. The MC count significantly decreased passing from adjacent NM towards C tissue [median 79.15 (range 22.5–192.7) *versus* 56.3 (15.8–97.8), respectively; *P* < 0.0001, by Mann–Whitney test]. Moreover, a statistically higher median expression of PAR-2 was observed in C compared with NM [2.7 (0–17.7) *versus* 26.5 (6.7–62.0), respectively; *P* < 0.0001].

**Fig. 2:**
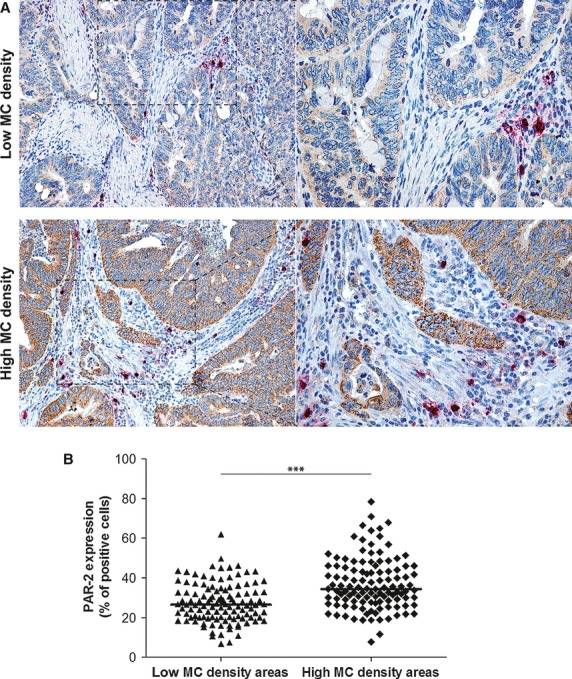
Comparison of PAR-2 expression in low and high mast cell density tumour areas. (**A**) Two representative images of PAR-2 immunoreactivity in colonic tumour areas with low and high MC counts by immunohistochemical double staining (original magnification on the left ×100, enlargement on the right ×200). (**B**) The distribution of PAR-2(+) cells, evaluated on the whole section with low MC density and at the sites where MCs most intensively accumulated in tumour lesion. (Horizontal bold line in each box = median value; ***P < 0.0001).

PAR-2 expression was evaluated both on the entire section and at the sites where MCs most intensively accumulated in serial sections (Fig. 2). Intriguingly, we noted a more distinct immunoreactivity (Fig. 2A) and a statistically higher median value of PAR-2 at the sites most infiltrated by MCs than in areas with low density of MCs [34.4 (7.7–78.4) *versus* 26.5 (6.7-62.0), respectively; *P* < 0.0001; Fig. 2B).

### Protein expression analysis of NHERF1

**Fig. 3:**
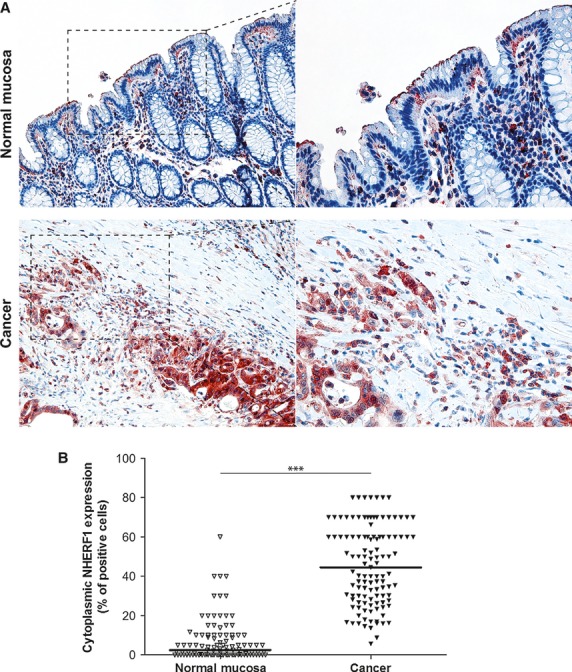
Expression analysis of cytoplasmic NHERF1 in human colorectal cancer. (**A**) Representative images of cytoplasmic NHERF1 immunoreactivity in the primary tumour matched with adjacent cancer-uninvolved colonic mucosa by immunohistochemistry (Original magnification on the left ×100, enlargement on the right ×200). (**B**) The distribution of cytoplasmic NHERF1(+) cells on normal mucosa and the tumour compartment of the same colonic lesion. (Horizontal bold line = median value; ***P < 0.0001).

In normal mucosa, NHERF1 expression was detected at the apical pole of the well-polarized duct, with a characteristic distribution along apical cell membranes of both enterocytes and goblet cells (Fig. 3A, upper panel). In the cells of all tumour samples, a variable cytoplasmic NHERF1 expression was detected. Positive cells had no preferential distribution. In fact, they could be observed either along the invasion front or within the growing tumour mass (Fig. 3A, lower panel).

A higher density of epithelial cells positive for cytoplasmic NHERF1 was observed in C compared with NM [44.5 (5.7–80.0) *versus* 2.5 (0–60.0) respectively] and the intergroup comparison showed that the median expression of NHERF1 was statistically higher in the C compartment than in NM (*P* < 0.0001; Fig. 3B).

### Association of clinicopathological data with MC density, PAR-2 and NHERF1 expressions

Significant associations between tumour markers and clinicopathological characteristics are summarized in Table 2. In particular, a high density of tryptase(+) MCs was shown in 48.7% (*n* = 56/115) of tumours, exhibiting a statistical association with male patients (*P* = 0.004) and higher TNM stage (*P* = 0.025); a statistical trend was observed between the high density of tryptase(+) MCs and tumours with a poor differentiation grade (*P* = 0.051).

**Table 2:** Association between tumour marker expressions and clinicopathological data in 115 colorectal cancers

Characteristics, *n* (%)	MC density		PAR-2 expression		Cytoplasmic NHERF1 expression	
		
	Low	High	Negative	Positive	Negative	Positive
Gender						
Male	26 (39.4)	40 (60.6)	31 (47.0)	35 (53.0)	32 (48.5)	34 (51.5)
Female	33 (67.3)	16 (32.7)	30 (61.2)	19 (38.8)	26 (53.1)	23 (46.9)
*P* value	**0.004**		NS		NS	
TNM stage						
I + II	17 (45.9)	20 (54.1)	23 (62.2)	14 (37.8)	28 (75.7)	9 (24.3)
III	20 (74.1)	7 (25.9)	21 (77.8)	6 (22.2)	23 (85.2)	4 (14.8)
IV	22 (43.1)	29 (56.9)	17 (33.3)	34 (66.7)	7 (13.7)	44 (86.3)
*P* value	**0.025**		**0.000**		**0.000**	
Differentiation grade						
High	6 (85.7)	1 (14.3)	5 (71.4)	2 (28.6)	5 (71.4)	2 (28.6)
Moderate	31 (54.4)	26 (45.6)	37 (64.9)	20 (35.1)	39 (68.4)	18 (31.6)
Poor	22 (44.0)	28 (56.0)	19 (38.0)	31 (62.0)	14 (28.0)	36 (72.0)
*P* value	**0.051**		**0.005**		**0.000**	
LVI						
Negative	34 (56.7)	26 (43.3)	42 (70.0)	18 (30.0)	44 (73.3)	16 (26.7)
Positive	10 (35.7)	18 (64.3)	11 (39.3)	17 (60.7)	8 (28.6)	20 (71.4)
*P* value	NS		**0.010**		**0.000**	
EGFR						
Negative	23 (46.9)	26 (53.1)	25 (51.0)	24 (49.0)	28 (57.1)	21 (42.9)
Positive	12 (66.7)	6 (33.3)	10 (55.6)	8 (44.4)	5 (27.8)	13 (72.2)
*P* value	NS		NS		**0.053**	

MC density: density of Tryptase-positive Mast Cell; PAR-2: Proteinase-Activated Receptor 2; NHERF1: Na^+^/H^+^ exchanger regulating factor 1; NS: not significant; LVI: lymphovascular invasion.

Significant associations (*P* < 0.05) are indicated in bold.

Positive expression of PAR-2 was observed in 47% (*n* = 54/115) of tumours and these overexpressed cases showed a significant association with high nodal status, the presence of distant metastases (*P* = 0.007 and *P* = 0.002, respectively, data not shown), higher TNM stage (*P* = 0.000), poor differentiation grade (*P* = 0.005) and the presence of LVI (*P* = 0.010).

Tumours overexpressing cytoplasmic NHERF1 (49.6%) showed a significant association with pT3-pT4 depth of invasion, high nodal status, the presence of distant metastases (*P* = 0.000 for all, data not shown), higher TNM stage (*P* = 0.000), poor differentiation grade (*P* = 0.000) and the presence of LVI (*P* = 0.000). Finally, tumours overexpressing cytoplasmic NHERF1 showed a significant trend with positive EGFR status (*P* = 0.053).

### Correlation among MC density, PAR-2 and NHERF1 expressions

**Fig. 4:**
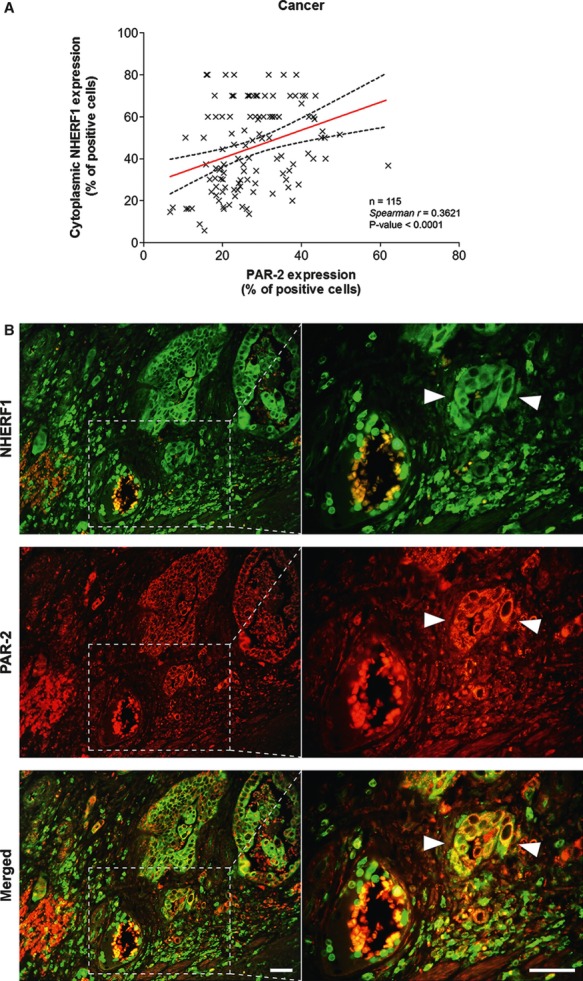
Analysis of PAR-2 and cytoplasmic NHERF1 in human colorectal cancer. As shown in (**A**), the correlation between protein expression of PAR-2 and cytoplasmic NHERF1 was evaluated by Spearman's rank correlation coefficient analysis, and a positive significant correlation was established. (**B**) A representative tissue sample stained with PAR-2 and EBP-50 antibodies and detected with Alexa Fluor 568 (red) and Alexa Fluor 488 (green) secondary antibodies, respectively, prior to fluorescence microscopy analysis. Overlaps between red and green signals (merged) point to co-localizations (in yellow). Arrowheads indicate invasive cells disseminated into the stroma close to a blood vessel with a high global expression of two proteins, where PAR-2 co-localized with NHERF1 on cytoplasmic and membranous compartments (scale bar = 20 μm).

A direct association existed between high MC density and PAR-2 overexpression. In fact, the Spearman's rank test revealed a positive correlation between tryptase(+) MCs and PAR-2 both in NM (*r* = 0.299, *P* = 0.002; Table 3B) and C compartments (*r* = 0.193, *P* = 0.039; Table 3A). A direct association existed between PAR-2 overexpression and increasing levels of NHERF1. The Spearman's rank test showed a significant positive correlation between PAR-2 and cytoplasmic NHERF1 both in NM (*r* = 0.319, *P* = 0.001; Table 3B) and C compartments (*r* = 0.362, *P* < 0.000; Fig. 4A). This association was also observed in immunofluorescence analysis, showing a co-overexpression of the two proteins in C samples. Tumour cells disseminated in the stroma and, frequently, very close to blood vessels within the tumour microenvironment, overexpressed PAR-2 and NHERF1, showing a wide co-localization of the two proteins in the cytoplasm compartment (Fig. 4B).

**Table 3:** Correlation analysis among Mast Cell density, PAR-2 and NHERF1 expressions in primary cancer (A) and paired normal colonic mucosa (B)

Variables	MC density	PAR-2	Cytoplasmic NHERF1
(A)			
MC density			
Rho	1	0.1931	0.167
*P* value	–	**0.039**	0.074
PAR-2			
Rho	0.1931	1	0.3621
*P* value	**0.039**	–	**<0.000**
Cytoplasmic NHERF1			
Rho	0.167	0.3621	1
*P* value	0.074	**<0.000**	–
(B)			
MC density			
Rho	1	0.2989	0.3485
*P* value	–	**0.002**	**0.000**
PAR-2			
Rho	0.2989	1	0.3191
*P* value	**0.002**	–	**0.001**
Cytoplasmic NHERF1			
Rho	0.3485	0.3191	1
*P* value	**0.000**	**0.001**	–

MC density: density of Tryptase-positive Mast Cell; PAR-2: Proteinase-Activated Receptor 2; NHERF1: Na^+^/H^+^ exchanger regulating factor 1.

Significant correlations (*P* < 0.05) are indicated in bold.

Given the fundamental association between these two tumour markers, we examined the potential prognostic role of the PAR-2/NHERF1 immunophenotypes. With respect to the PAR-2(−)/cytoplasmic NHERF1(−) expression phenotype, the PAR-2(+)/cytoplasmic NHERF1(+) phenotype was associated with the highest depth of invasion (*P* = 0.004), positive nodal status (*P* < 0.000), the presence of distant metastases (*P* < 0.000), poor differentiation grade (*P* < 0.000) and the presence of LVI (*P* = 0.000; Table 4).

**Table 4:** Association between PAR-2/NHERF1 expression immunophenotypes and clinicopathological data

Characteristics, *n* (%)	PAR-2(+)/cytoplasmic NHERF1(+)	PAR-2(−)/cytoplasmic NHERF1(−)	*P* value
Depth of invasion			
pT1	0 (0.0)	3 (100.0)	**0.004**
pT2	2 (14.3)	12 (85.7)	
pT3	28 (57.1)	21 (42.9)	
pT4	7 (58.3)	5 (41.7)	
Nodal status			
pN0	7 (21.9)	25 (78.1)	**<0.000**
pN1	10 (41.7)	14 (58.3)	
pN2	17 (89.5)	2 (10.5)	
pN3	3 (100.0)	0 (0.0)	
Distant metastases			
M0	8 (20.0)	32 (80.0)	**<0.000**
M1	26 (92.9)	2 (7.1)	
Differentiation grade			
Well	1 (20.0)	4 (80.0)	**<0.000**
Moderate	13 (28.9)	32 (71.1)	
Poor	22 (75.9)	7 (24.1)	
LVI			
Negative	10 (21.7)	36 (78.3)	**0.000**
Positive	13 (76.5)	4 (23.5)	

PAR-2: Proteinase-Activated Receptor 2; NHERF1: Na^+^/H^+^ exchanger regulating factor 1; LVI: lymphovascular invasion.

Significant associations with *P* < 0.05 are indicated in bold.

Furthermore, a significant correlation was highlighted between high MC density and increasing levels of cytoplasmic NHERF1 in the NM compartment (*r* = 0.349, *P* = 0.000; Table 3B).

## Discussion

Although a variety of malignant tumours are accompanied by an increased infiltration of MCs, conflicting data about the relationship between MC density and prognosis in CRC have been reported. In fact, although some authors have found a direct correlation between high MC counts and improved prognosis [26, 27], the majority of studies have shown that MCs are directly associated with an unfavourable prognosis and tumour aggressiveness [28–31]. In this study, we investigated the distribution of MCs and their relationship with PAR-2(+) epithelial cells in primary CRC. Using the immunohistochemical double staining technique, we analysed the density of tryptase(+) MCs and the expression of PAR-2 in tumours and adjacent uninvolved colonic mucosa.

In this study, we showed a large distribution of MCs in the lamina propria and submucosa, representing an early and persistent infiltrating immune cell type. In line with findings from Xia *et al*. [32], we confirmed that the MC count in normal mucosa was higher than that in primary tumours. Interestingly, we also demonstrated a significant MC increase in male patients compared with female patients in agreement with Wu *et al*. [33]. This strong association seems to be related to the oestrogen/oestrogen receptor/tumour necrosis factor-α axis; in fact, MCs express oestrogen receptors and treatment with the specific ligand has been demonstrated to prevent the release of TNF-alpha, a major MC-derived autocrine growth factor [34].

In our study, MCs infiltrated stroma more abundantly at invasive margins than in the internal tumour field, probably to activate cell processes involved in cancer progression. Furthermore, we showed that tumours with distant metastases and with a poor differentiation grade had a higher density of tryptase(+) MCs. Tryptase(+) MCs have been demonstrated to play a role in tissue remodelling during wound healing and cancer, degrading selectively matrix proteins, synthesizing collagen [35] and stimulating fibroblast proliferation [36] and myofibroblast differentiation [25]. However, it has been suggested that in the context of developing tumours, the ability of MCs to remodel tissues is subverted, disrupting the surrounding extracellular matrix and increasing tumour spread [37]. In normal colonic mucosa, natural inhibitors of tryptase and normal tissue homeostasis maintain a proteolytic balance, even if during cancer progression, this balance is gradually disturbed and definitively broken in the invasive CRC by overexpression of several proteases [38]. On the basis of our results, it seems that MCs might functionally contribute to neoplastic aggressiveness by secreting tryptase, which increases tumour invasion and metastasis [39, 40].

**Fig. 5:**
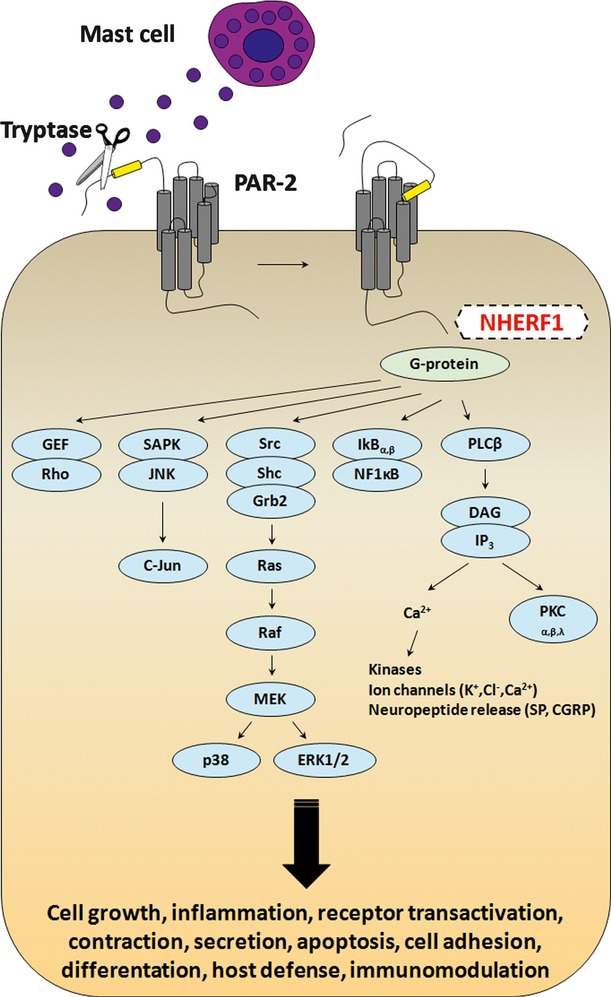
Diagram showing the major G protein–mediated signalling pathways coupled to PAR-2. Tryptase secreted in the tumour microenvironment by infiltrating mast cells cleaves the N-terminal domain of PAR-2, releasing a new N-terminal tail, which acts as a tethered ligand that binds the receptor itself. The association with G proteins initiates signal transduction, resulting in stimulation of phosphoinositide breakdown, cytosolic calcium mobilization and promoting several cell responses.

Recent studies have altered the traditional view of the role of proteases in tumour evolution, showing that these enzymes serve as signal molecules controlling cell functions through specific membrane receptors [41]. In this context, it has been suggested that PAR-2 may play an active role in the setting of cancer growth and tumour progression (Fig. 5) [42–47]. Furthermore, most colon cancer cell lines express PAR-2 and its activation has shown to play an important role in the progression of colon cancer [10]. In our study, we demonstrated an increased expression of PAR-2 in cancer with respect to adjacent normal mucosa and PAR-2 overexpression was associated with advanced disease parameters, as measured by TNM stage, poor histological differentiation and LVI. Furthermore, we described a positive linear correlation between MC density and PAR-2 expression, hypothesizing that tryptase may act directly on PAR-2 by stimulating cancer progression. In fact, analogous results have been reported for trypsin, a serine proteinase similar to tryptase, which regulates cellular proliferation in gastric carcinoma [48] and promotes colon cancer proliferation by inducing PAR-2/G protein signalling pathways (Fig. 5) [10]. Thus, our data could support the view that PAR-2 expression is related to the progression of CRC and its tryptase-induced activation could have a role in tumour invasion.

Mounting evidence highlights the role of cytoplasmic adapter proteins that contribute critically to the signalling, trafficking and function of many G protein–coupled receptors [49]. In our previous studies, it was established that NHERF1, an adaptor protein with two non-identical, type 1 tandem PDZ domains and a carboxyl-terminal ezrin-binding domain, is a potential candidate of clinical relevance for human breast cancer [16, 24, 50, 51]. Recently, we have also demonstrated that nuclear NHERF1, present in the early stages of colorectal carcinogenesis and related with poor prognosis, might contribute to the onset of the colonic malignant phenotype [19]. Furthermore, we have suggested nuclear NHERF1 as a potential new biomarker of advanced CRC, demonstrating an overexpression of nuclear NHERF1 in no longer polarized epithelial cells, converted to a mesenchymal phenotype in hypoxic colonic areas [20]. Therefore, given that the role of nuclear NHERF1 has been previously analysed in-depth and considering that reports investigating the distribution of cytoplasmic NHERF1 in CRC have not been published to date, in this study, we aimed to analyse the role of the NHERF1 protein localized in the cytoplasm. We demonstrated that overexpressing tumours resulted associated with unfavourable prognosis and aggressive clinical parameters. Moreover, we showed both a positive linear correlation between cytoplasmic NHERF1 and PAR-2 and a significant co-expression of the two proteins mostly in the margin of the tumour mass.

Even if PAR-2 expression was associated with clinicopathological characteristics of adverse prognosis, in some studies, PAR-2 has not represented an independent prognostic factor [52, 53]. Other markers interacting with the PAR-2 signalling pathway may be the reason of this trend, which influences survival outcome. Thus, study of PAR-2 expression in CRC in combination with other proteins could be helpful in evaluating the relationship of PAR-2 with patient survival [52]. We therefore examined the potential prognostic role of PAR-2/NHERF1 immunophenotypes on the basis of the significant correlation between these two tumour markers. Interestingly, the PAR-2(+)/cytoplasmic NHERF1(+) expression immunophenotype was able to predict poor prognosis of CRC patients, being associated with the presence of nodal and distant metastasis, poor differentiation grade and LVI.

In conclusion, our findings show that the high density of tryptase(+) MCs correlates with the advanced stages of CRC. Furthermore, the strong association between tryptase(+) MCs and PAR-2 expression seems to suggest that the activation of tryptase-induced PAR-2 contributes to tumour progression and invasiveness. Furthermore, examination of the PAR-2(+)/cytoplasmic NHERF1(+) expression immunophenotype appears able to predict the prognosis of CRC and may be useful for selecting subgroups of patients who should be treated with more aggressive therapies because of the higher risk of tumour invasion and metastasis.

Finally, we believe that further studies are needed to confirm usefulness of PAR2(+)/cytoplasmic NHERF1(+) expression immunophenotype for selecting subgroups of patients who should be treated with more aggressive therapies.
